# Shame, stigma, HIV: philosophical reflections

**DOI:** 10.1136/medhum-2016-011179

**Published:** 2017-08-08

**Authors:** Phil Hutchinson, Rageshri Dhairyawan

**Affiliations:** 1 Faculty of Health, Psychology and Social Care, Manchester Metropolitan University, Manchester, UK; 2 Department of Integrated Sexual Health, Barking and Dagenham, Havering and Redbridge University Hospital Trust, Outpatients East, Manchester, UK

**Keywords:** Shame, Stigma, HIV, Philosophy

## Abstract

It is a distinctive feature of HIV that its pathology cannot be adequately grasped separate from a number of psychosocial factors, and stigma is widely seen as the most prominent. We argue that it is equally important to have an adequate understanding of shame, as the emotional response to stigma. We have identified five ways shame might negatively impact upon attempts to combat and treat HIV, which emerge from the stigma HIV carries and STI-stigma in general. In this paper, we draw out four insights from philosophical work on emotions and shame which we propose will improve understanding of shame and stigma. We conclude by briefly discussing how these insights might shed light on the negative role shame can play for a person living with HIV engaging with, or being retained in, care. We conclude by proposing further study.

It is a distinctive feature of HIV that its pathology cannot be adequately grasped separate from a number of psychosocial factors, and stigma is the most prominent of these. Of the literature on the topic of HIV stigma, Parker and Aggleton^1^ have produced arguably the most influential recent work, in which they argue for a hybrid social theory of stigma, which while indebted to Goffman’s classic study of stigma, seeks to augment this by drawing on Foucault’s analysis of power. We propose, with qualifications, that the present paper be seen as a critical companion piece to Parker and Aggleton’s paper. Where their focus is on stigma, ours is on shame. Where they are concerned to enrich understanding of stigma in the context of HIV, so we seek to do likewise with shame.

However, here is where we would wish to qualify our endorsement of Parker and Aggleton’s paper: First, we propose that to examine stigma without also examining shame is to miss much of importance; it is akin to studying ‘threats’ as a social phenomenon, while ignoring the fear people have in response to those threats. Second, while the thrust of Parker and Aggleton’s argument is well taken and important, it fails to get to the important conceptual questions, because it remains at the level of social theory. We argue below that philosophical reflection on shame can offer significant gains, which can inform effective strategies for care. Such gains are to be found below the philosophical waterline^i^ and serve to frame more overtly empirical and theoretical considerations of emotion, shame and stigma. Our paper aims to be a reminder that mitigating, and where possible dissolving, conceptual confusion through philosophical work is crucial if one is to do good psychological and sociological work.

We have identified five ways in which shame might negatively impact upon attempts to combat and treat HIV, which emerge from the stigma HIV carries and STI-stigma in general.

1. Shame can prevent an individual from disclosing all the relevant facts about their sexual history to the clinician.
2. Shame can serve as a barrier to engaging with or being retained in care.
3. Shame can prevent individuals presenting at clinics for STI and HIV testing.
4. Shame can prevent an individual from disclosing their HIV (or STI) status to new sexual partners.
5. Shame makes people want to hide and withdraw from the world and others, it therefore makes the task of living with HIV a far more negative experience than it should, or needs to, be.

In concluding this paper, and by way of example, we will focus on No. 2. We will propose that our four insights, gained from philosophical work on emotions and shame, provide guidance for future study of the negative role shame might play for a person living with HIV engaging with or being retained in care.

## Philosophical Reflection on Emotions and Shame

Explanations of emotion fall in to one of two philosophical camps. Those whose work falls within the paradigm initiated by the 19th-century philosopher, psychologist and physician William James, are referred to as Jamesians.^ii^ In short, you are a Jamesian if you argue that emotions are essentially physiological responses to causal stimuli, and the awareness of these responses. Emotion as affect.

In opposition, one finds the cognitivists, a position in the philosophy of emotions traceable back to the Stoic movement in Ancient Greece, but with contemporary adherents in contemporary cognitive psychology, ecological cognition and postanalytic philosophy. You are a cognitivist if you argue that emotions are essentially thoughts about, or directed towards, something in the world. Emotion as (embodied) thought.^iii^

One could advance one of many psychological or sociological theories of emotion without referring to either of these two underlying philosophical positions, but in doing so, in advancing your theory, you will commit to one of them, and this commitment would then constrain you. One is so constrained because whether acknowledged or not, these philosophical commitments will be operative in one’s theorising.

This philosophical dispute has consequences for addressing, or treating emotions, where they present difficulties. If we conceive of emotions as essentially physiological changes triggered by causal stimuli in the environment, our focus will be on understanding the causal mechanisms, and perhaps preventing the cause. On this view, the person experiencing the emotion is *subject to* that emotion, the emotions can be depicted as ‘passions’ and a person who is in an emotional state is passive.

Depicting the emotions as passions leads us to depict the person having the emotion as akin to a stimulus-response processor, responding to certain causal inputs with certain outputs (emotional responses). While this has the virtue of being a naturalistic depiction of emotion, it is somewhat at odds with the complexity of human psychology and mind–world relations; it also faces the somewhat difficult problem of accounting for the meaning emotions have for those experiencing them. On this view, we understand human emotional responses on the model of an artefact such as a smoke alarm responding to smoke in the environment.

If one is a cognitivist, the picture is different, a little more complex, and, we propose, more appropriate to our nature. Here, our emotions are meaningful responses to a world which we experience as having meaning for us, what in some philosophical contexts is referred to as the lifeworld. On this conception, emotions are no longer appropriately depicted as passions and the bearer of emotions is no longer necessarily passive but, rather, has scope for some emotional agency, for it is at least logically (or grammatically) possible to achieve control over one’s emotions, if not necessarily possible in practice, on specific occasions.

Within the cognitivist approach to explaining emotion there are a number of different sets of commitments one might have, which relate to the nature of the cognitive constituents one employs in the explanation. So, one might be a judgementalist (eg, Solomon^2^), where one depicts emotions as constituted and type individuated by judgements. Here one is also, therefore, committed to a representationalist picture of cognition, where the constituents of cognition are mental representations of the world, with propositional form. On this view, the emotion is a propositional attitude. So, where judgementalists identify the constituents of emotion as judgements, other philosophers identify the cognitive constituents as ‘thoughts’ (Nussbaum^3^), ‘evaluative beliefs’ (Taylor^4^) or ‘construals’ (Roberts^5^); we can, therefore, refer to this group of emotion theorists as ‘propositional attitude cognitivists’ (Pro-Cogs), for they are all committed to the claim that the constituents of emotion are propositional attitudes. Pro-Cogs conceive the person as a belief (or judgement) machine, akin to software programmes running on hardware components, which output contentful information in response to certain patterned inputs.

In contrast, and at the other end of the cognitivist spectrum, we find those who forgo or reject representationalism, and depict emotions as a specific class of affordances or evaluative perceptions (eg, Hutchinson^6^). We call this version of cognitivism ecological cognition (Eco-Cog), and the explanation becomes less theoretical and abstract and instead focused on persons who have gone through a process of enculturation, who inhabit a world which has meaning and contains loci of significance for them, given their enculturation (Bildung/Second Nature) and their particular interests.^iv^ While this framework forgoes brevity and neatness, perhaps, we gain in understanding and explanatory power, for it is sensitive to the way in which the world has meaning, and the specific meaning it has for this person might be in response to that person’s enculturation and interests.

We here want to pick out two gains we believe arise from taking up the Eco-Cog perspective on emotion.^v^

1. An emotion is not conceived of as an affect (Jamesianism), nor is conceived of as constituted by beliefs (Pro-Cog). Rather, we conceive of emotion as a dynamic relation between, on the one hand, a person, who in addition to their biological constitution is endowed with a specific culture, and on the other hand, the lifeworld. This means that theoretical solutions will not cut it, because the generality of the theory will mean it is unable to capture the dynamism and occasion, context and cultural specificity of the relationship. A *theory* of shame will fail to adequately capture the nature of the emotion. Rather, we must engage with the individuals experiencing the emotion and work to understand the way in which they relate to the world and how that brings them to the emotional state.
2. The approach we recommend overcomes the ‘problem of emotional recalcitrance’ (see D’Arms and Jacobson^7^ and Griffiths^8^), oft-cited as a reason to forgo Pro-Cog in favour of Jamesianism: an example of emotional recalcitrance being *fear* of flying, experienced while concurrently believing (correctly) that flying is the safest mode of transport. On the Eco-Cog approach, belief is not playing the explanatory role which it plays in Pro-Cog, so, we can retain the cognitivist insight without facing the problem of recalcitrance. This pays dividends when we turn our attention to shame, for it is a characteristic feature of shame that it is experienced in the absence of a set of beliefs that would serve to explain the shame. Indeed, often it is the converse: the beliefs held tell the person experiencing shame that they have no grounds for their shame, yet the shame persists.

## Emotions and Their Objects: The World-Directed Nature of Emotion

Cognitivist explanations of emotion depict emotions as object oriented: emotions have objects, and the conceptual analysis of emotion, by which we establish their identity criteria, includes these objects. On this account, shame’s object is status diminishment, of one’s self (one’s being), as an individual or as a member of a group. To be stigmatised, as an individual or member of a stigmatised group, is to be subject to such a diminishment in status. Shame is the emotional manifestation of such stigma. In philosophical terms, shame and stigma are *internally related*, for they are not related as two separate entities but are internal to the meaning and identity of each other.

Let us take some examples from the writings of Primo Levi, and his discussions of shame in *If this is a Man* 9 and later in *The Drowned and the Saved*.^10^ We do so because, as we noted above, our analysis should be focused on people expressing and experiencing the emotions we are studying.

Primo Levi’s writings testify to a number of sources of status diminishment that give rise to shame:

- Humanity was diminished because the crimes of Auschwitz had taken place: so humanity bore the stigmata engendered by the crimes of the Holocaust (the bureaucratised and industrialised dehumanisation, mass murder and genocide). Levi’s shame testifies to this stigma.
- In addition, Levi felt diminished owing to the accident of survival. He experiences shame because survival was not connected to his worth as a person, because death and survival in Auschwitz were radically decoupled from one’s goodness or badness, or any discernible rationale, becoming a matter of chance, decided by the whim of a psychopath or perverse bureaucratic system. This diminishes the status of the individual, post-Auschwitz. It seems that for Levi his survival felt unearned, and while others died, this amounts to a diminishment.
- There also seems to be a diminishment which comes from a mismatch between how one acted in Auschwitz—even allowing for judging these as irreproachable in the context of the camp—and the standards one sets for oneself, or that are reasonably expected of one by one’s honour group, in the pre and post-Auschwitz context. Here, what is stigmatised is the person acting as they did in the camp, viewed from the perspective of the postcamp culture, and the recognition that the viewer and viewed are the same person (or group).

There are a number of things that we can learn from these examples that are central to philosophical discussions of shame and that, we will argue, are relevant to HIV-related shame and stigma. We here want to focus on stigma-as-object-of-shame.

## Emotions and Objects

Our first observation is that while emotions have objects, these need not be concrete, but can be diffuse and abstract. The object of fear might well be the bear standing before us on the mountain path, though even here, it is our *taking*, our evaluative perception, of the bear-as-a-threat-to-our-continued-wellbeing and the conceptual resources that draws upon, that constitute it as an object of fear. However, this said, the object of our fear in this example is there before us in very real and literal sense. In contrast, we might also experience fear of failure, and here the object of fear is the idea of our failing to achieve that which is either expected of us by our peers or by ourselves. While the bear has a kind of concreteness and is directly perceived in the act of fearing, the future failure exists only as a possible future outcome, and as such is neither concrete nor directly perceptible.

Returning to shame; as we saw in the first example taken from Levi’s writings, what had become stigmatised was ‘humanity,’ and Levi experiences shame as he acknowledges this, for his identity is in part constituted by his own humanity—not only his membership of the species *Homo sapiens*, but how ‘humanity’ as a moral concept, what affords us the evaluative terms humane and inhumane, for example, features in our identity. Humanity is stigmatised owing to the crimes of the Holocaust and individual members of humanity experience shame in acknowledging this stigma and their membership of the stigmatised group.

So here is gain 3:

3. Objects can be concrete, simple and directly perceived or diffuse, complex and indirectly conceived. If the unfolding of history can stigmatise the very concept of humanity bringing shame for Holocaust survivors like Levi, then this tells us something about how some acts become stigmatised over time, through political change, for example, and lead to shame where there was none before.

## Shame, Honour Groups, Autonomy and Heteronomy

Discussions of the role of audience or honour group have been prominent in philosophical work on shame^5 7 17^ and the related question as to whether shame is an essentially heteronomous or autonomous emotion (eg, refs. 6 11–13). The question is this: does the negative evaluation of others, taking the role of audience or honour group, play an essential role in the emergence of an individual’s shame? If one answers ‘yes,’ then one is implying in that answer that shame is heteronomous: shame is dependent on the judgement of others. If one answers ‘no,’ then one allows that shame can be autonomous: shame is not dependent on the judgement of others. This question cuts to the heart of how we might address shame.

Shame can appear to be characteristically heteronomous, as we see in the kind of shame that an individual experiences when they have fallen short of some societal standard. Consider the third in the list of shame experiences we find in Levi’s writings. Here Levi experienced shame because his actions in Auschwitz did not meet the standards expected of individuals in post-Auschwitz, post-War, European society. This is an example of heteronomous shame, because the shame emerges from the mismatch between a standard which is external to the individual, which resides in an actual audience evaluating the individual’s action or in a social rule or norm. Both actual audience and social norm provide a standard against which the person’s status is diminished.

Alternatively, one might argue that shame is essentially autonomous, where shame comes from a mismatch between one’s prevailing assumptions about one’s own character and the more considered, honest, reflective judgements made about that character. We might seek to project an image of our character as morally upstanding, caring and sharing. But, when we take time to reflect, when we are honest with ourselves about the totality of our behaviour over time, we see a mismatch and we see we are not the person we assumed ourselves to be and who we sought to project to others. This is an example of autonomous shame.

This discussion has persisted in the philosophical literature on shame because it cuts to the heart of our moral psychology. From the perspective of (modern) morality, if shame were to be essentially heteronomous then it can play no significant role in an individual’s moral psychology, because to be a moral agent demands that one act on one’s own moral judgements, even when these are at odds with the judgements of the honour group or established social norms. If shame is to be an emotion worthy of a place in analyses of our moral psychology then it cannot merely track the dominant social attitudes.

We draw on Hutchinson’s^6^ arguments to propose that shame can appear to operate, from case to case, either autonomously or heteronomously, but that ultimately the distinction collapses. For a person to take an event as shameful is for them to have read it as such, for it to have had that meaning *for them. That* person, *this individual,* has acknowledged the event as shameful. Looked at this way, shame meets the criteria for being autonomous.

However, there are certain enabling conditions that are required for this autonomy. The world has meaning for individuals because individuals go through processes of enculturation, which include the learning of a (public) language; in turn, learning a language is to learn to register, acknowledge and communicate the loci of significance in our world. In this sense, we can see the heteronomous sources of shame. The event is shameful because it has that meaning for us. It has that meaning in virtue of our reading it this way and thereby *seeing it as* shameful. What enables our reading of the world in this way, seeing it under this aspect, is our second nature—our character—which is formed in and through our life with others.

While shame is intimately related to our social, encultured nature, it is therefore a mistake to assume this makes it necessarily heteronomous in a way which disqualifies it from having moral significance. Understanding that shame is not necessarily heteronomous, but can be autonomously arrived at should not be to deny that the social sources of our enculturation, the development of our moral character, serve as enabling conditions for our life with shame.

Shame *can* be heteronomous, such that shame is an acknowledgement, a taking-on-board, of the judgements (or morally loaded perceptions) of others about one’s self, and in so doing considering oneself, one’s being, to be in some way evaluatively diminished. While shame *can* also be autonomous, such that shame serves as testament to a mismatch between the sense of self one assumes and seeks to project to others and the self that one considers oneself to be on reflection.

Insisting it must be one or the other has consequences: if one holds that shame is always autonomous, then that will lead one to focus any attempt to alleviate shame solely on the psychology of the individuals who bear shame. Conversely, if one were to assume that shame is always heteronomous, then that will lead one to identify that which is in need of change as being the social norms of which the honour group (the shame-instantiating audience) are an embodiment. In this latter, heteronomous sense, addressing shame might be a political, cultural and social task, in addition to being a psychological task. This is our fourth gain.

4. We propose that addressing and treating shame can be, and often is, a political, cultural *and* psychological task.

### Staging Post: Four Insights from Philosophical Work on Emotions and Shame

Let us here summarise our four gains, which we believe emerge when one dips below the philosophical waterline and engages with the philosophy of emotion and shame.

1. Emotional responses should be understood from the perspective of *Ecological Emotional Cognition*, as having the structure of a dynamic relation between an encultured individual with interests and the constitution of the lifeworld as a world of meanings and affordances. Theoretical accounts will not work. We must engage with the individuals experiencing the emotion and work with them to understand the way in which they relate to their world and how that brings them to the emotional state.
2. The Eco-Cog approach has the resources to make sense of someone having an emotion, while concurrently believing their emotion unwarranted. This is a characteristic feature of shame, where often the person experiencing shame believes there are no grounds for their shame.
3. Emotions have objects, but these objects need not be simple, concrete nor directly perceptible. Shame’s object is stigma, and the stigma can take the form of a diffuse, complex of attitudes, which are taken not via perceptual capacities but which might be conveyed by such things as conceptual metaphor and familiar objects having gained new symbolic status.
4. At one level, shame experiences might be either heteronomous or autonomous, depending on the particular experience. At a deeper level, shame is the product of an individual’s evaluative taking of loci of significance in the lifeworld, which in turn are enabled by that individual’s enculturation. We propose that responding to shame will therefore demand political change in addition to work on the individual’s psychological health.

### Shame, Stigma and HIV: Retention in Care

HIV serves as a vector through which pass many of a society’s existing prejudices. It is this that we identify as HIV-stigma. The shame experienced by some people living with HIV has as its object HIV-stigma. HIV-stigma can emerge from many sources, which track wider social attitudes. Since HIV is widely thought of as a sexually transmitted infection, HIV-stigma can draw upon prejudicial and other negative attitudes to sex and sexuality; where attitudes prevail that identify certain sexual acts as normal, and others as abnormal or perverse, certain sexualities as normal and others as abnormal or perverse, the latter become stigmatised. HIV-stigma might even be related to attitudes about the appropriate amount of sex or number of sexual partners an individual should have, and perhaps this in turn invokes further gendered prejudice. Certain groups within society might have come to be more readily associated with HIV than are others and existing prejudices regarding these groups might therefore become associated with an HIV diagnosis.

Our focus on existing prejudices regarding sex and sexuality should not lead us to overlook the extent to which HIV-stigma might sometimes draw upon HIV’s status as a chronic illness (at least in those places where Anti-Retroviral Therapy (ART) is readily available), and how living with chronic illness and the consequent dependence on health resources can lead to various sources of stigma: stigma associated with society’s emphasis on good health, on autonomy, where your dependency on health services, on medication and the constant reminder that you do not have good health, can serve to stigmatise. The stigma might even emerge from metaphors widely employed to talk about viral infection, which introduce by stealth negative moral appraisals. Here, as we comment elsewhere,^14^ the question ‘are you clean?’ asked of a friend might well have been innocent and meant no intentional stigmatisation, but the effect might serve to provide an object for shame.

Currently, as there is no known cure for HIV, successful treatment of HIV involves taking antiretroviral medication daily for the rest of an individual’s life (adherence), and attending HIV services regularly (retention in care). This is known to be challenging in many chronic diseases, such as diabetes and hypertension, where good adherence is thought to be >60% of drug doses taken correctly; it is even more challenging in HIV, where in order to avoid drug resistance >95% of drug doses need to be taken correctly.

Episodic treatment interruptions cause problems. First, there is some evidence to suggest that they increase drug resistance, where a person whose ART regimen is interrupted responds less well to their ARVs as a result. Second, breaks from treatment increase the chance of a patient’s health deteriorating, their developing clinical AIDS or even of their death. The SMART (Strategies for Management of Antiretroviral Therapy) trial, involving just short of 5500 volunteers in 33 countries comparing continuous with episodic treatment of HIV, was stopped early as it became clear that patients following episodic treatment were at twice the risk of developing clinical AIDS.^15^ While treatment in many countries has improved since the SMART trial ended in 2006, this cannot be claimed to be the case globally; the results of the trial are relevant for many people living with HIV. Third, breaks in treatment can lead to spikes in viral load and therefore much greater risk of onward transmission.

While there are many possible factors that might lead to the interruption of care, from physical barriers, socioeconomic problems, and so on, the stigma associated with HIV, and the shame that emerges from this, are widely understood to be significant contributory factors in many decisions to stop or take a break from treatment, and it is this with which we are concerned here. What we here want to do, therefore, is twofold:

- motivate the take-up of further study through which we might better understand the role shame and stigma play in the challenge posed to adherence and retention in care;
- provide the conceptual resources so we that we can undertake sound research, employing methods appropriate to the nature of our topic: shame and HIV-stigma.

While, from a narrowly conceived biomedical perspective, ART might well enable an individual to live a normal life, from a psychosocial perspective that same therapy—the taking of the pills, the attending clinic, the blood tests—might serve to continually remind the same person of their HIV status and the stigma associated with HIV. The very thing that biochemically suppresses the virus can serve, psychosocially, to generate stigma and shame.

### How Might this be Addressed?

Following the first of our four gains, we propose studies which enable our understanding of the shame experienced by people living with HIV, particularly those who have taken a break from treatment, so that we might better understand the objects of the emotion and the conditions under which those objects ‘come into view.’ We propose that facilitating studies conducted by people living with HIV themselves, studies conducted in accordance with ethnographic principles and techniques provided by Ethnomethodology,^vi^ would be the most productive approach.

Following our second gain, treatment strategies designed to mitigate the effects of the emotion or eradicate it should not be based on explaining to a person that they have no good reason to be ashamed of their HIV status; those individuals will be likely to know this, they might even believe this strongly and be able to cite clear supporting reasons. Given what we know about the conceptual anatomy of shame, we need to address ourselves to the emotion at the subpropositional level.

Following our third gain, and following on from first and second, we might set about understanding the specific nature, the genesis and the conceptual anatomy of HIV-stigmas. The genesis will vary, but will be likely to have political sources in both intentional stigmatising practices, such as discriminatory laws, and acts of discrimination, and in unanticipated consequences of well-meaning interventions such as public health messaging, which targets certain groups or practices. It is also likely that there will be residual structural stigmatisation, as the laws are repealed and the public health messaging improved, which resides in the metaphors still present in the language. Prejudicial language lingers long after prejudicial laws have passed into history and even after attitudes have largely changed. Such language can stigmatise. Again, ethnographies designed to identify the source, production and maintenance of stigma will bring forth the best insights here.

Our fourth gain serves to help us guard against the pathologising of those who experience shame. Their shame experience is often a response to the lifeworld and where support needs providing it should not be exclusively focused on the psychological health of the individual: we need to transform social practices where those serve to generate or perpetuate shame. It is important that psychological support is offered where it can be of help, but psychological support alone in the absence of the required political and societal changes amounts, overall, to a failure of support. Giving support in the shape of psychotherapy while leaving untouched the policy decisions that generate stigma is to take a crassly irresponsible approach to psychological well-being.

## Conclusion

We have identified four insights that emerge from a philosophical work on emotions and shame. We have sought to relate these to the problem of HIV-stigmas and shame, as that impacts upon adherence and retention in care for people living with HIV. Our proposal is that this work of philosophical reflection guides us in further approaches to much needed study.

## Footnotes

i What we intend to draw out by employment of this metaphor is the extent to which philosophy is non-theoretical activity of conceptual clarification and dissolving of conceptual confusions. This is work that needs to be done, and which cannot be bypassed by simply producing more theory or doing more empirical work. To do this work, one must dip below the philosophical waterline.

ii This is sometimes referred to as the James-Lange theory, because the Danish physiologist Carl Lange arrived at the same theory at around the same time.

iii See Hutchinson^16^ for a more detailed overview of Jamesianism and Cognitivism.

iv There is an ongoing discussion in Philosophy of Mind, Psychology and Cognitive Science, and which we report on here because it impacts directly on our conception of emotion; the argument is over whether cognition is essentially representational. There is a tradition which has dominated for much of the last one hundred years in analytic philosophy, and in cognitive science for the last fifty years, which sees the constituents of cognition (thought) as essentially mental representations of the world, which have semantic content. In contrast, there are accounts which challenge this representationalist picture. These one can find in Ecological and Enactivist accounts of cognition (eg, Gibson,^17^ Noe,^18^ and Hutto & Myin^19^) and Ethnomethodological accounts (eg, Coulter^20^). Such accounts often draw on philosophical work by Ludwig Wittgenstein and by Maurice Merleau-Ponty.

v For those who are familiar with Dan Moerman’s^21^ writing on meaning responses, those writings can be usefully seen as a companion to that which we propose here. Moerman argues that stimulus-response (Pavlovian conditioning) and response-expectancy (belief and expectation) ‘explanations’ fail to explain the ‘placebo response’, and in their place proposes a non-representationalist meaning response, which invokes our encultured nature.

vi Ethnomethodology, as an approach to social inquiry, is concerned with how social norms and rules are generated, maintained and repaired through the interaction and communication of members of society. Moreover, ethnographic descriptions of these interactions should give priority to the concepts employed by the participants. The most effective way to ensure adherence to these principles, so that we might gain an insight in the norms which govern the distribution of stigma and shame within the social settings which people living with HIV inhabit, is to provide the resources so that people living with HIV can themselves produce ethnographic descriptions of, for example, a visit to the dentist, or the GP, or attending a new clinical setting. For further reading on Ethnomethodology, see Garfinkel^22^ and Button (ed.)^23^
